# Targeting the Cerebrospinal Fluid Compartment in Glaucoma: Still the Dark Side of the Moon? Comment on Passaro et al. Glaucoma as a Tauopathy—Is It the Missing Piece in the Glaucoma Puzzle? *J. Clin. Med.* 2023, *12,* 6900

**DOI:** 10.3390/jcm13030827

**Published:** 2024-01-31

**Authors:** Peter Wostyn

**Affiliations:** Department of Psychiatry, PC Sint-Amandus, 8730 Beernem, Belgium; wostyn.peter@skynet.be; Tel.: +32-472713719; Fax: +32-50-819720

I read with great interest the article by Passaro et al. [1] exploring the novel perspective of glaucoma as a tau-associated disorder and shedding light on potential new therapeutic avenues for this disease through targeting various pathogenic pathways involved in tauopathies. I would like to congratulate the authors for establishing this informative review and would appreciate the opportunity to make a comment.

The authors provide an interesting overview of potential therapeutic strategies for tauopathies, and possibly glaucoma, including the suppression of microtubule-associated protein tau expression, the regulation of alternative splicing, the stabilization of microtubules, the regulation of post-translational modifications, the inhibition of aggregation, the activation of tau clearance, the use of passive or active immunization, and innovative approaches related to genome integrity preservation. They also comprehensively discuss the structural changes to the eye that link Alzheimer’s disease (AD) and glaucoma. Several ocular abnormalities, such as a reduction in the number of optic nerve head axons, a reduced number of retinal ganglion cells (RGCs), and a thinning of the retinal nerve fiber layer (RNFL) and RGC layer, have been demonstrated in AD, and are also early hallmarks of glaucoma. As further noted by the authors, several studies have revealed an increased occurrence rate of glaucoma among AD patients. They also discuss a recent study by Lee et al. [2], which investigated the relationship between the lamina cribrosa’s thickness and cognitive function in glaucoma patients. This study found that a thinning of the lamina cribrosa was significantly associated with worse cognitive function. Lee et al. [2] suggested that cognitive impairment and glaucomatous damage share a common mechanism of lamina cribrosa thinning. Based on the above observations and the findings of a study by Lee et al. [3] discussed below, I believe that there may be a scientific rationale for targeting the cerebrospinal fluid (CSF) compartment to prevent or delay glaucomatous optic nerve damage.

Although there are intriguing overlaps between AD and glaucoma, some links need to be further explored to achieve a deeper understanding. Lee et al. [3] investigated whether the RNFL thinning observed in AD patients could be explained by the relationship between abnormal CSF profiles and their influence on the lamina cribrosa’s thickness. It has been shown that the lamina cribrosa is thinner in glaucoma patients than in healthy controls [4]. A thinner lamina cribrosa is associated with a steeper translaminar pressure gradient (intraocular pressure (IOP)–intracranial pressure/thickness of the lamina cribrosa) and may be more susceptible to deformation [3,4]. Under these conditions, axonal transport can be blocked, contributing to the development of RGC damage [3,4]. Lee et al. [3] reported that a higher CSF level of total tau (t-tau) was associated with a thinner lamina cribrosa both in healthy subjects and in AD patients. According to the authors, one possible explanation for this observation could be that the accumulation of tau causes a collapse of the cytoskeleton and the degeneration of laminar astrocytes, leading to the increased susceptibility of astrocytes to degradation, resulting in further thinning of the lamina cribrosa [3]. Interestingly, in a previous report, Nucci et al. [5] described a glaucoma patient with medically controlled IOP who experienced disease progression concomitantly with the onset of mild cognitive impairment and positivity for CSF markers of AD (decreased amyloid-beta 42 (Aβ42) and elevated levels of t-tau and phosphorylated tau). Nucci et al. [5] suggested the possibility that altered CSF circulatory dynamics in this case reduced neurotoxin clearance along the optic nerves in the subarachnoid space (SAS) and that deposits/aggregates of tau and/or other toxic molecules may have contributed to the glaucoma’s progression.

Emerging evidence suggests a potential role for altered CSF dynamics in glaucoma [6,7]. Impaired CSF dynamics within the SAS of the optic nerve have been demonstrated in normal-tension glaucoma (NTG) [6,7]. These findings suggest that the SAS surrounding the optic nerve can develop into a separate CSF compartment with a different CSF composition, in the setting of NTG [6]. If altered CSF composition affects the pathophysiology of glaucoma, it would be worthwhile to further explore whether strategies for removing toxic substances from the CSF might be effective treatment approaches for glaucoma. In this context, approaches targeting CSF dynamics could be new directions for glaucoma treatment. Such interventions could improve CSF circulation around the optic nerve, leading to the enhanced removal of potentially neurotoxic waste products that accumulate in the optic nerve. Given that such interventions might reduce the CSF levels of tau and/or other toxic substances in the SAS surrounding the optic nerve, this might protect against the thinning of the lamina cribrosa and subsequent glaucomatous optic nerve damage. It is also interesting to note that the accumulation of amyloid precursor protein and Aβ in the optic nerve has been demonstrated in mouse models of glaucoma [8]. It has been postulated that glaucoma, just like AD, may be the result of an imbalance between the production and clearance of neurotoxins, including Aβ [9]. Interestingly, in mice, Mathieu et al. [10] found evidence that CSF enters the optic nerve via a glymphatic pathway, a CSF transport system that facilitates the clearance of neurotoxic molecules, including Aβ, through a network of paravascular pathways. The authors found paravascular CSF entry into the optic nerve up to and including the glia lamina, the mouse equivalent of the human lamina cribrosa. They suggested that CSF flow through the optic nerve may play a role in neurotoxin clearance in the laminar and retrolaminar optic nerve, with potential implications for the pathogenesis of glaucoma [10]. In a more recent article, Mathieu et al. [11] provided evidence that CSF entry into the optic nerve SAS and optic nerve paravascular spaces is impeded following tracer injection into the CSF in a DBA/2J mouse model of glaucoma. If, indeed, CSF is involved in the clearance of solutes and wastes, including Aβ, from the optic nerve, then strategies to regulate glymphatic clearance could offer new therapeutic approaches to glaucoma. I therefore wish to encourage further research in this area.
